# The Urinary Bladder Transcriptome and Proteome Defined by Transcriptomics and Antibody-Based Profiling

**DOI:** 10.1371/journal.pone.0145301

**Published:** 2015-12-22

**Authors:** Masato Habuka, Linn Fagerberg, Björn M. Hallström, Fredrik Pontén, Tadashi Yamamoto, Mathias Uhlen

**Affiliations:** 1 Science for Life Laboratory, KTH - Royal Institute of Technology, SE-171 21, Stockholm, Sweden; 2 Department of Immunology, Genetics and Pathology, Rudbeck Laboratory, Uppsala University, SE-751 85, Uppsala, Sweden; 3 Department of Structural Pathology, Institute of Nephrology, Medical and Dental School, Niigata University, 1-757, Asahimachi-dori Niigata, 951-8510, Japan; 4 Department of Proteomics, KTH - Royal Institute of Technology, SE-106 91, Stockholm, Sweden; University of Glasgow, UNITED KINGDOM

## Abstract

To understand functions and diseases of urinary bladder, it is important to define its molecular constituents and their roles in urinary bladder biology. Here, we performed genome-wide deep RNA sequencing analysis of human urinary bladder samples and identified genes up-regulated in the urinary bladder by comparing the transcriptome data to those of all other major human tissue types. 90 protein-coding genes were elevated in the urinary bladder, either with enhanced expression uniquely in the urinary bladder or elevated expression together with at least one other tissue (group enriched). We further examined the localization of these proteins by immunohistochemistry and tissue microarrays and 20 of these 90 proteins were localized to the whole urothelium with a majority not yet described in the context of the urinary bladder. Four additional proteins were found specifically in the umbrella cells (Uroplakin 1a, 2, 3a, and 3b), and three in the intermediate/basal cells (KRT17, PCP4L1 and ATP1A4). 61 of the 90 elevated genes have not been previously described in the context of urinary bladder and the corresponding proteins are interesting targets for more in-depth studies. In summary, an integrated omics approach using transcriptomics and antibody-based profiling has been used to define a comprehensive list of proteins elevated in the urinary bladder.

## Introduction

The main function of the urinary bladder is to store the urine made by the kidneys, allowing urination voluntarily [1], a process regulated by the nervous system. Urinary bladder consists of adventitia, muscularis propria and urothelium. Urothelium plays an important role in preventing rupture of urine storage during bladder distention and of intercellular junctions for leaking of toxic urinary substances into the blood. The urothelium consists of three to seven layers of umbrella cells, intermediate cells and basal layer cells. Umbrella cells are superficial and elliptical cells having abundant eosinophilic substance, mucin in the cytoplasm. An interesting undertaking is to increase our knowledge of the molecular functions of the urinary balder under physiological and also pathological conditions by characterization of the proteins expressed in the cells comprising the different parts of the urinary bladder. Previously, molecular biology studies, such as positional cloning, in situ hybridization and immunohistochemistory, have been performed to discover and characterize new urinary bladder proteins and their functional roles. Uroplakin (UPK) was discovered to form urothelial plaques on the apical surface of the urothelium [2] and keratin 5 (KRT5) in the urothelial stem cells and progenitor cells locating in the KRT5+ basal layers [3]. Yang et al identified urinary proteins as biomarkers of urinary bladder cancer by a proteomic approach using nano-HPLC ESI-MS/MS technology, and confirmed their results by Western blotting [4], while Zhang et al recently identified cancer recurrence-related genes in human urinary bladder tissues by transcriptomic approach [5]. Despite these advances in our knowledge, a comprehensive urinary bladder-specific transcriptome and proteome has not yet been defined. Recently, Kim et al presented the proteomic profiling of the human proteome in various human tissues, but the urinary bladder was not included as one of the tissues in the report [6].

Here, we decided to carry out a comprehensive genome-wide analysis of the human urinary bladder tissue using transcriptomics (RNA-seq) including tissue types representing all major tissues and organs in the human body [7, 8]. This analysis was combined with an immunohistochemistry (IHC) analysis to localize the protein gene products at single cell level. Since the quantitative analysis using transcriptomics is performed in mixtures of cell types of urinary bladder tissues, the subsequent immunohistochemistry analysis provided spatial information in the different compartments of the bladder. In this manner, we have generated a knowledge resource with a comprehensive list of genes elevated in urinary bladder with data on specificity and precise spatial distribution of the corresponding proteins in the urinary bladder.

## Materials and Methods

### Transcript profiling (RNA-seq)

The two tissue samples used for transcript profiling of human urinary bladder were selected as histologically normal tissue from operated materials. The use of human tissue samples was approved by the Uppsala Ethical Review Board (Ups 02–577, no. 2011/473). The use and analyses based on human tissues has previously been described in Fagerberg L et al [8] Tissues samples were embedded in Optimal Cutting Temperature (O.C.T.) compounds from Sakura, Japan, and stored at –80°C. Frozen sections (4 μm) were prepared from each sample in a cryostat using the CryoJane^®^ Tape-Transfer System (Instrumedics, St. Louis, MO, USA). To ensure proper tissue morphology sections were stained with hematoxylin-eosin (HE) and examined by a pathologist as normal. For RNA extraction, three sections (10 μm) were cut from each tissue block. Tissue was homogenised using a 3-mm steel grinding ball (VWR). Total RNA was extracted using the RNeasy Mini Kit (Qiagen, Hilden, Germany) according to the manufacturer’s instructions. RNA samples were analysed using either an Agilent 2100 Bioanalyser system (Agilent Biotechnologies, Palo Alto, USA) with the RNA 6000 Nano Labchip Kit or an Experion automated electrophoresis system (Bio-Rad Laboratories, Hercules, CA, USA) with the standard-sensitivity RNA chip. All samples had an RNA integrity number of > 7.5. RNA seq (mRNA sequencing was performed on Illumina HiSeq2000 and 2500 machines (Illumina, San Diego, CA, USA) using the standard Illumina RNA-seq protocol with a read length of 200 bases.

### Analysis of data

Sequencing of samples from 32 tissues and organs was performed using Illumina HiSeq2000 and 2500 as previously described [7, 8]. Briefly, Tophat v2.0.8b [9] was used to map processed reads to the human genome (GRCh37). FPKM (fragments per kilobase of exon model per million mapped reads) values were calculated using Cufflinks v2.1.1 [10], which corrects for transcript length and the total number of mapped reads from the library to compensate for different read depths for different samples. The gene models from Ensembl build 75 were used in Cufflinks [11] and the total number of genes with transcript expression data was 20,344. All data was analysed using R Statistical Environment (http://www.r-project.org/) and network analysis was performed using Cytoscape version 3.0 [12]. Where a log2-scale of the data was used for analyses, pseudo-counts of +1 were added to the data set. The significance of the enriched/enhanced genes were calculated using the DESeq software [13], doing a pairwise comparison between all the biological replicates of the enriched/enhanced tissue (or group of tissues) and all other tissues. The multiple-testing adjusted P-value (FDR 5%) was used to determine the significance of the enrichment.

### Data availability

All the data (FPKM values for all the samples) are available to download from the Human Protein Atlas website (www.proteinatlas.org/about/download). The primary data (reads) are available through the Array Express Archive (www.ebi.ac.uk/arrayexpress/) under the accession number E-MTAB-2836. The transcript profiling data (FPKM values) for each gene in each tissue is available in the version 13 of the Human Protein Atlas (www.proteinatlas.org).

### Specificity classification

An average FPKM value of all individual samples for each tissue was used to estimate the gene expression level [8]. A cut-off value of 1 FPKM was defined as the detection limit. Each of the 20,344 genes were classified into one of seven categories based on the FPKM levels: (1) “Not detected in any tissue”—< 1 FPKM in 32 tissues; (2) “Not detected in urinary bladder”—< 1 FPKM in urinary bladder; (3) “Bladder enriched”—5-fold higher FPKM level in urinary bladder compared to all other tissues; (4) “Group enriched”– 5-fold higher average FPKM level in a group of 2–7 tissues including urinary bladder compared to all other tissues; (5) “Bladder enhanced”– 5-fold higher FPKM level in urinary bladder compared to the average FPKM value of all 32 tissues; (6) “Expressed in all tissues”–detected in 32 tissues >1 FPKM; (7) “Mixed”–genes expressed in 1–31 tissues and in none of the above categories.

### Tissue profiling

Tissue microarrays (TMA) containing triplicate 1-mm cores of 46 different types of normal tissue and duplicate 1-mm cores of 216 different cancer tissues representing the 20 most common forms of human cancer were generated as previously described [14]. All of the tissues used as donor blocks were acquired from the archives at the Department of Pathology of Uppsala University Hospital in agreement with approval from the Research Ethics Committee at Uppsala University (Uppsala, Sweden) (Ups 02–577). TMA sections were immunostained using antibodies evaluated in the HPA project [15]. The AperioScanScope XT Slide Scanner (Aperio Technologies, Vista, CA) system was used to capture digital whole slide images with a 20X objective. Slides were de-arrayed to obtain individual cores. The outcome of the TMA IHC stainings in the screening phase, was manually evaluated by certified pathologists and scored using a web-based annotation system (unpublished). In brief, the manual score of IHC-based protein expression was determined as the fraction of positive cells defined in different tissues: 0 = 0–1%, 1 = 2–25%, 2 = 26–75%, 3>75% and intensity of immunoreactivity: 0 = negative, 1 = weak, 2 = moderate and 3 = strong staining. The immunohistochemical staining was visually evaluated with regard to urinary bladder specificity and cellular distribution allowing further determination of protein localization within different sub-compartments/cell types in the urinary bladder.

## Results

### The transcriptome of urinary bladder tissue

**Fig 1:**
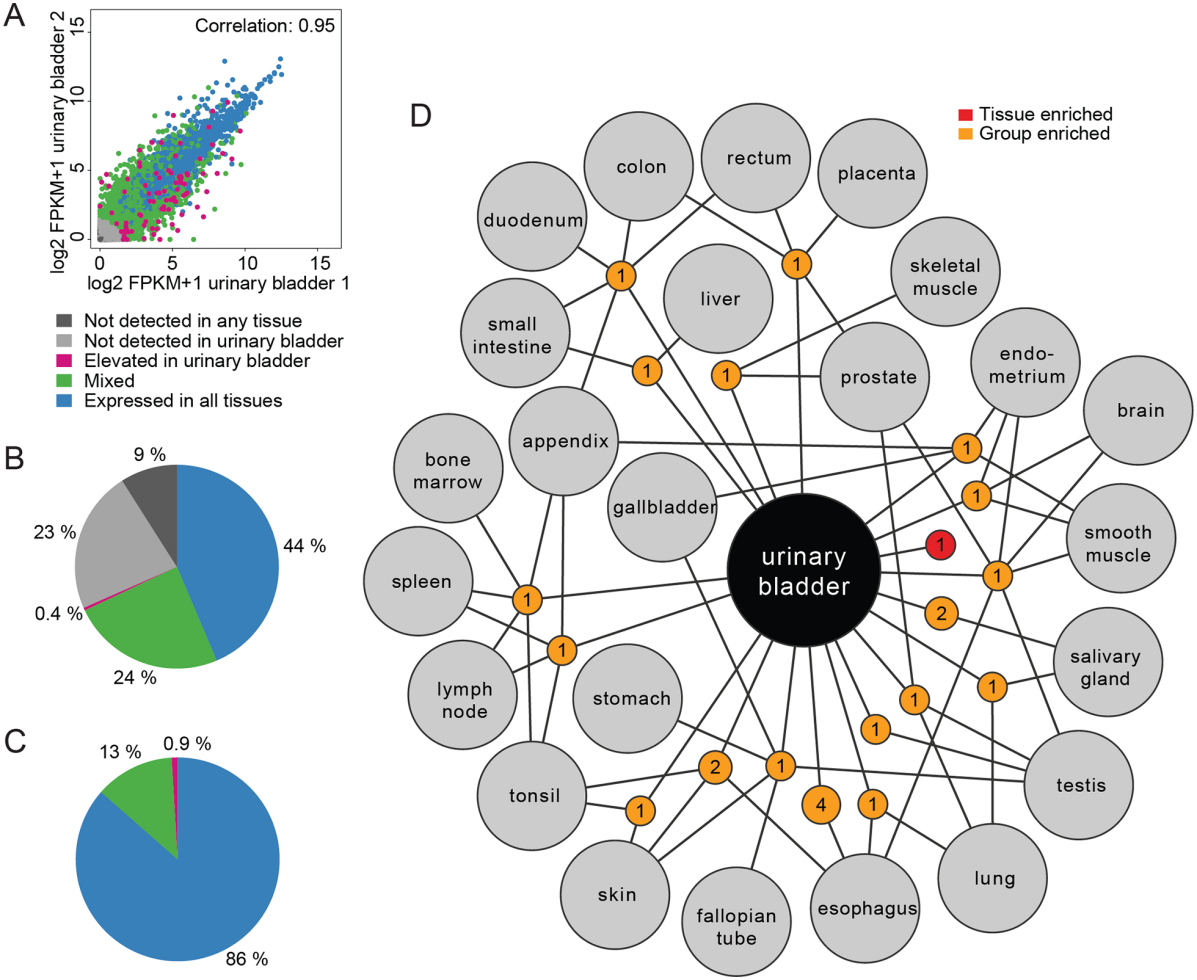
Classification of all protein-coding genes based on their expression in human urinary bladder tissue. (A) Scatterplot showing the FPKM values of all 20,344 protein-coding genes in the two urinary bladder samples, with each gene colored according to category. (B) Pie chart showing the distribution of all protein-coding genes into five categories based on transcript abundance and number of detected tissues, including expression in all 32 tissues (blue), mixed expression with genes expressed in a varying number of tissue (green), genes with elevated expression in urinary bladder (pink), not detected in urinary bladder (light grey), and not detected in any tissue (dark grey). The genes with elevated expression in urinary bladder are further subdivided depending on the degree of specificity as tissue enriched genes, group enriched genes and tissue enhanced genes in urinary bladder (Table 1). (C) Pie chart showing the distribution of the fraction of expressed mRNA molecules, i.e. the sum of all FPKM values for each of the categories for the genes expressed in urinary bladder, using the same color codes. (D) Network plot of the urinary bladder enriched gene (red) and group enriched genes (orange). Orange circle nodes represent a shared group of expressed genes and are connected to the respective enriched tissues (grey circles). The size of each orange node is related to the square root of the number of genes enriched in a particular combination of tissues.

We performed deep sequencing (RNA-seq) analysis on two fresh frozen urinary bladder samples and analyzed our results against transcriptomics data collected from 31 other tissue types including all other major organs based on specimens from altogether 122 individuals [8]. The transcriptome of each sample was quantified to determine the normalized mRNA levels, calculated as FPKM, where one FPKM roughly corresponds to one mRNA molecule per average cell in the sample [16]. Histological analyses of all specimens were performed to verify that cell type compositions of samples included were representative of the respective tissue. In the two analyzed urinary bladder samples, the approximate constitution was: urothelial cells (5–10%), smooth muscle cells (45–60%), fibroblasts (15–20%), endothelial cells (10%) and other cell type (5–20%) as shown on the Human Protein Atlas for a description in selected samples (http://www.proteinatlas.org/ENSG00000105668-UPK1A/tissue/urinary+bladder). Of all human putative protein coding genes (n = 20,344), expression of a total of 13,914 genes was detected in the urinary bladder, with a mean expression value of 1l FPKM with a cut-off of 1 FPKM (in average approximately 1 mRNA molecule per cell). This cut-off was chosen based on earlier suggestions [8]. Thus, approximately 68% of all putative protein-coding genes were detected in the urinary bladder. The variations between the two individual urinary bladder samples were analyzed by calculating the pairwise Spearman correlation (0.95) and plotting the expression level (FPKM values) of all 20,344 protein-coding genes (Fig 1A). As expected, pairwise comparisons between the urinary bladder and the 31 other tissue types showed a higher variance. The lowest correlation was found between urinary bladder and testis (0.71) while gallbladder displayed the highest similarity (0.95) to urinary bladder (data not shown).

### Classification of the genes expressed in urinary bladder

The transcriptomics data obtained from 32 different tissue types enabled us to classify all putative protein-encoding genes into five categories (Fig 1B) based on their expression levels in urinary bladder. Almost half (44%; n = 8,874) of genes were expressed in all analyzed tissues and 24% (n = 4,954) of genes showed a mixed expression pattern, being expressed in a large subset of the tissues, including urinary bladder. A small fraction of genes (0.4%; n = 90) were found with an elevated expression in urinary bladder as compared to the other 31 tissues investigated and in the following we decided to investigate these further. 23% (n = 4,594) of genes were not detected in urinary bladder, but detected in at least one of the other tissues, while an additional 9% (n = 1,832) of the genes were not detected in any of the analyzed tissues, including urinary bladder. The 90 genes with elevated expression in the bladder were further divided into three categories (Table 1) based on degree of tissue specificity. Only one gene (UPK2) was defined as a highly enriched one with at least more than 5-fold higher mRNA levels in the urinary bladder compared to all other tissues. This finding was also supported by transcriptomics data generated within the GTEx consortium [17], http://www.gtexportal.org, where UPK2 was highly enriched in urinary bladder. In addition, 66 genes were defined as enhanced, with 5-fold higher mRNA levels in urinary bladder compared to the average FPKM value of all other tissues, and 23 genes were defined as group-enriched, with 5-fold higher average mRNA levels in a group of 2 to 7 tissues including urinary bladder (S1 Table). In Table 2, the 20 most tissue enhanced genes in the urinary bladder is listed.

**Table 1:** The number of genes with elevated expression in urinary bladder.

Expression Category	Description of increase in expression (FPKM) in urinary bladder by comparing to all other tissues	Number of genes
Tissue enriched	More than 5-fold higher expression exclusively in urinary bladder	1
Group enriched	More than 5-fold higher average values of expression in a group of 2–7 tissues including urinary bladder	23
Tissue enhanced	More than 5-fold higher in urinary bladder by comparing to the average expression value of all other tissues	66
Tissue elevated	Summation of all genes in the above categories All tissue enriched, enhanced and group enriched (total)	90

**Table 2:** The 20 most elevated genes in urinary bladder according to the transcriptome profiling.

Gene name	Description	Subcell prediction	Category	Bladder mRNA	TS Score
UPK2	uroplakin 2	TM	Bladder enriched	54	38.6
CLEC3A	C-type lectin	SP	Bladder enhanced	8	4.7
SNX31	sorting nexin 31	INTRA	Group—bladder, esophagus	26	4.6
SERPINB4	ovalbumin	INTRA	Group—bladder, esophagus	20	4.5
DHRS2	dehydrogenase	INTRA	Group—bladder, salivary gland	300	4.4
UPK1A	uroplakin 1A	TM	Group—bladder, esophagus	87	4.3
HOXA1	homeobox A1	INTRA	Bladder enhanced	8	4.0
UPK3B	uroplakin 3B	TM-SP	Group—bladder, esophagus	20	3.6
PADI3	deiminase	INTRA	Group—bladder, esophagus	19	3.4
DUOXA2	maturation factor	TM	Bladder enhanced	35	3.4
CYP24A1	cytochrome P450	INTRA	Bladder enhanced	81	3.3
DKK1	WNT signaling	SP	Bladder enhanced	22	3.0
KRT17	keratin 17	INTRA	Bladder enhanced	229	2.8
ALG1L	transferase	INTRA	Bladder enhanced	11	2.7
BMP3	bone protein	SP	Bladder enhanced	20	2.6
FSTL4	follistatin-like	SP	Bladder enhanced	15	2.4
TRPA1	transient receptor	TM	Bladder enhanced	28	2.4
FOXQ1	forkhead box Q1	INTRA	Bladder enhanced	31	2.3
EDARADD	EDAR-associated	INTRA	Bladder enhanced	26	2.3
CXCL5	chemokine ligand	SP	Bladder enhanced	16	2.3

Subcell prediction shows the predictive subcellular localization of each protein (TM; transmembrane, SP; secreted, TM-SP; overlap between TM and SP, and INTRA; intracellular) according to Uhlen et al [7, 36]. Bladder mRNA shows the average FPKM value in the urinary bladder samples. The genes have been order based on the tissue specific (TS) score calculated as the ratio between the FPKM value in urinary bladder divided by the maximum FPKM value of all other tissues.

Analysis of the expression levels of each gene in the urinary bladder made it possible to calculate the relative mRNA pool for each of the categories (Fig 1C). 86% and 13% of transcripts in this study of the urinary bladder corresponded to ‘housekeeping genes’ found in all analyzed tissues and ‘mixed’, respectively. On the other hand, only 0.9% of transcripts were categorized as either urinary bladder-enriched or group-enriched or enhanced. This demonstrates that only a minor number of transcripts correspond to protein-coding genes elevated in the urinary bladder.

An analysis of the group-enriched genes (Fig 1D and S2 Table) shows that urinary bladder shares many group-enriched genes with esophagus, as exemplified by PADI3, UPK1A and SNX31. This is not surprising since the urothelium, also known as transitional epithelium, is a stratified squamous epithelium, similar to the epithelium of the esophagus. In accordance with this, several of the group-enriched genes, for example UPK1A and SERPINB4, were also enriched in skin. UPK1A has been previously described as a gene expressed in esophagus and it has been shown to be down regulated in cancer tissue, both on mRNA and protein level [18]. SERPINB4 (SCCA2), also known as squamous cell carcinoma antigen 2, has been previously described as expressed in the suprabasal layers of normal stratified squamous, including esophagus [19], but it has not yet been described as elevated expression in urinary bladder. Similarly, PADI3 has been previously described with an elevated expression in skin [20], but here we show that the transcript is also elevated in urinary bladder and esophagus. Finally, SNX31, regulator of intracellular trafficking and signaling pathways, has not been previously described with elevated expression in urinary bladder. The above-mentioned group enriched genes were all supported by the GTEx data, except SERPINB4, which showed lower expression in urinary bladder than in seven other tissues.

### Antibody based profiling of the urinary bladder elevated genes

To further explore the genes with elevated expression in the urinary bladder, the protein localization in urinary bladder tissues was investigated using the data from the Human Protein Atlas (HPA) (www.proteinatlas.org) [21]. The HPA provides immunohistochemistry images of tissue microarray (TMA) cores from 46 different normal tissues. In the latest version (version 13), a total of 24,028 antibodies were used to evaluate the presence or absence of 16,975 proteins with the staining intensity. Here, the immunohistochemical staining was used to confirm urinary bladder specificity and to determinate protein localization within different sub-compartments and cell types. Out of the 90 genes that we identified as elevated in urinary bladder, 18 of the identified genes have not yet been analyzed in the HPA because of lack of antibodies for immunostaining, but these are interesting targets for future antibody-based profiling. The remaining 72 genes were investigated for localization in the urinary bladder and all were confirmed to be present in the urinary bladder using the antibody-based spatial profiling. In the following section, the protein localized to the different subcompartments of the urinary bladder will be discussed.

### Proteins localized in urothelium

**Fig 2:**
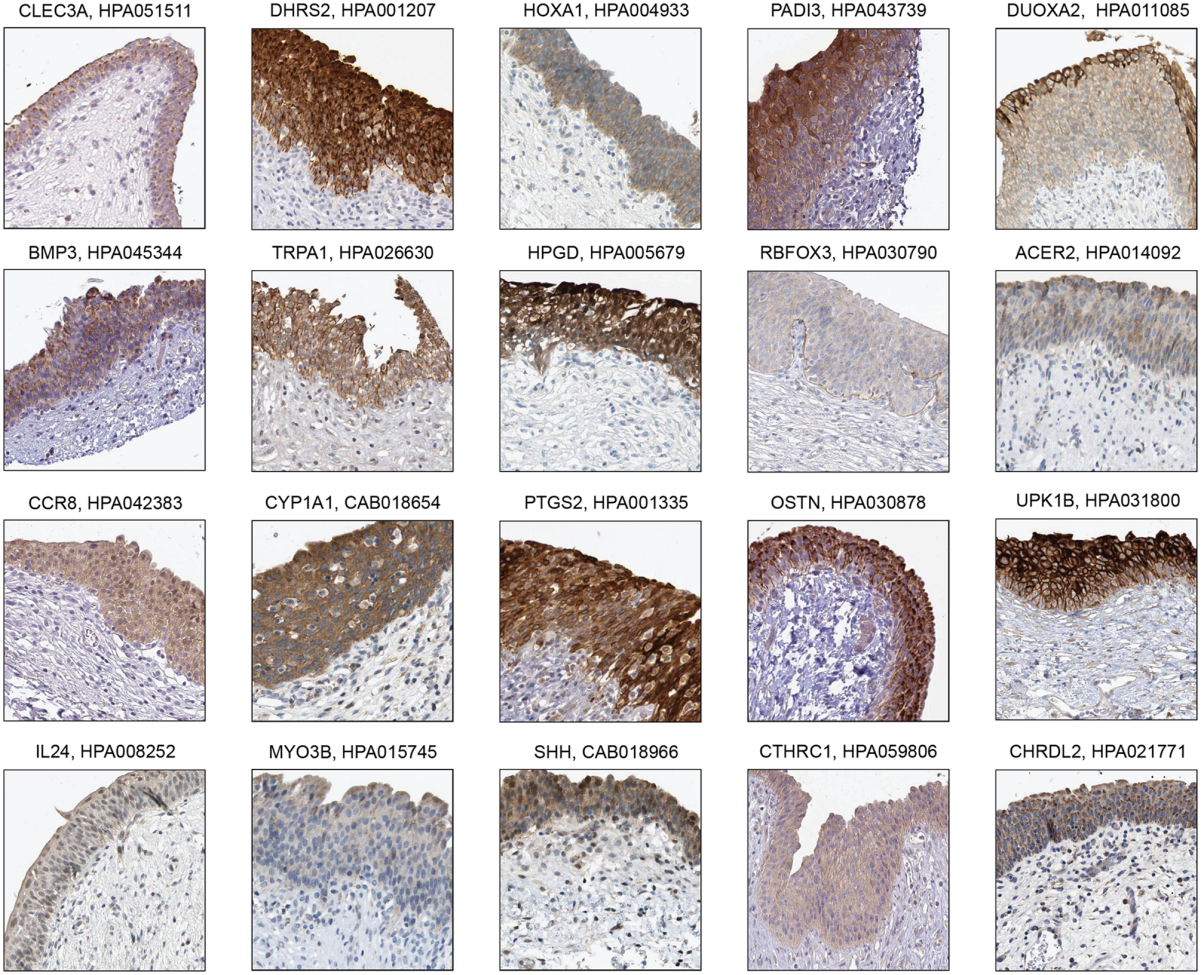
Immunohistochemistry-based protein profiling of elevated genes in urinary bladder that are expressed in urothelium. Of the 90 elevated genes in urinary bladder, 20 proteins are localized to the whole urothelium: CLEC3A (tissue enhanced gene), DHRS2 (group enriched gene), HOXA1 (tissue enhanced gene), PADI3 (group enriched gene), DUOXA2 (tissue enhanced gene), BMP3 (tissue enhanced gene), TRPA1 (tissue enhanced gene), HPGD (tissue enhanced gene), RBFOX3 (group enriched gene), ACER2 (tissue enhanced gene), CCR8 (tissue enhanced gene), CYP1A1 (group enriched gene), PTGS2 (tissue enhanced gene), OSTN (tissue enhanced gene), UPK1B (tissue enhanced gene), IL24 (group enriched gene), MYO3B (tissue enhanced gene), SHH (tissue enhanced gene), CTHRC1 (tissue enhanced gene), CHRDL2 (group enriched gene). All images are from The Human Protein Atlas and the title of each image shows the respective gene and antibody names.

Out of the 90 genes identified as elevated in urinary bladder, 20 were localized in the whole urothelium (Fig 2), and 14 out of the 20 genes, (CLEC3A, DHRS2, HOXA1, PADI3, DUOXA2, BMP3, RBFOX3, ACER2, CCR8, OSTN, IL24, MYO3B, CTHRC1, CHRDL2), have not been previously described in the context of the urinary bladder. Interestingly, some of the proteins encoded by the above-mentioned genes have previously been described as playing important roles in the proper outgrowth and patterning of the limb bud. The inhibition of bone morphogenetic proteins (BMPs) by BMP3 enhanced expression of fibroblast growth factors (FGFs) and sonic hedgehog (SHH), which are essential molecules for proper limb development [22] [23] [24] Moreover, HoxA also regulates the pathway [25]. SHH has been described as associated with the differentiation of smooth muscle in urinary bladder [26] and FGF10 as associated with not only the limb outgrowth, but also as a growth factor for bladder regeneration [27].

### Proteins localized in umbrella cells

**Fig 3:**
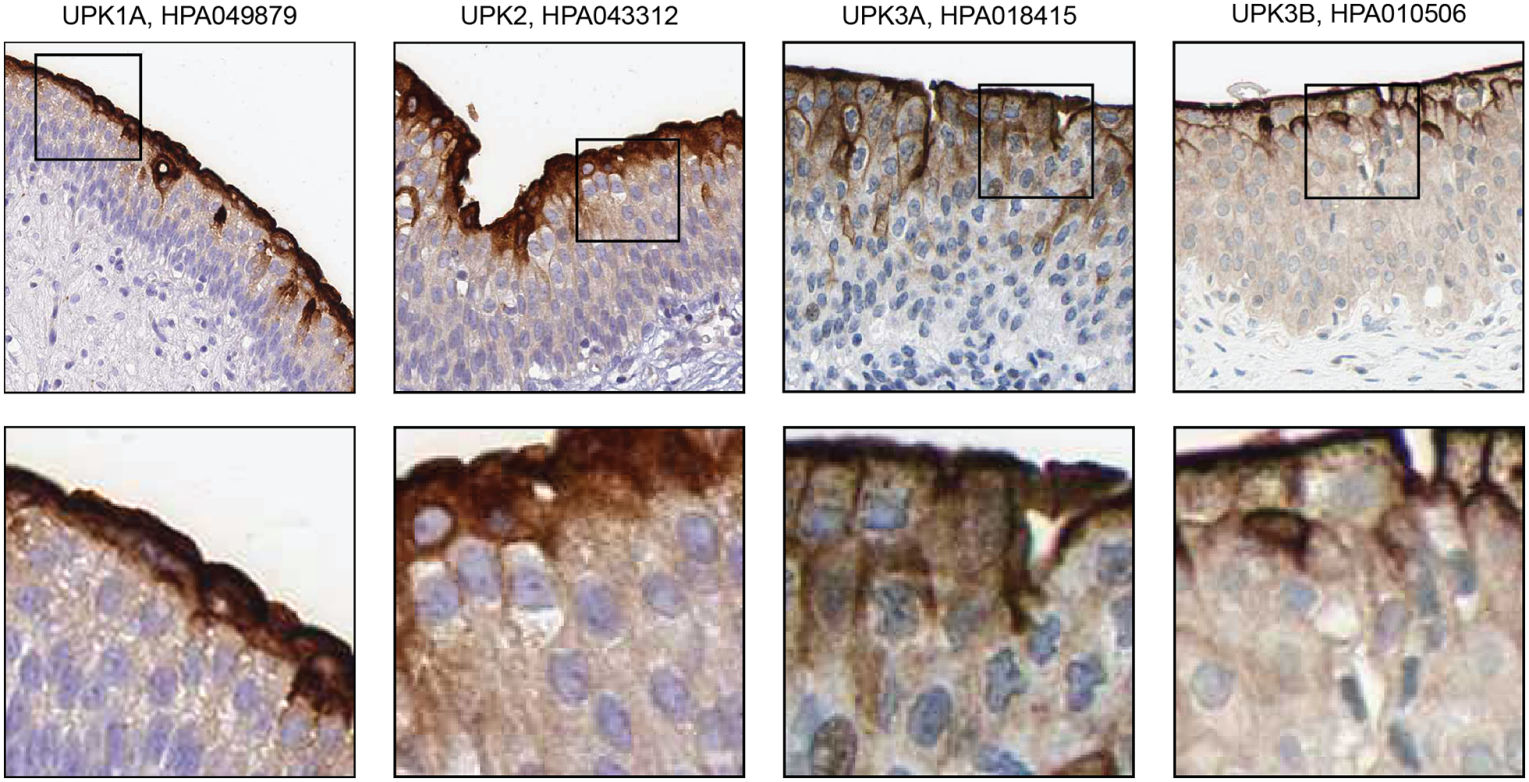
Immunohistochemistry-based protein profiling of elevated genes in urinary bladder that are expressed in umbrella cells. Of the 90 elevated genes in urinary bladder, UPK1A (group enriched gene), UPK2 (tissue enriched gene), UPK3A (group enriched gene) and UPK3B (group enriched gene) are localized specifically to umbrella cells in urothelium. All images are from the Human Protein Atlas and the title of each images show the respective gene and antibody names.

Out of the 90 genes identified as elevated in urinary bladder, four were found to be localized exclusively in the umbrella cells. These four all belong to the UPK family (uroplakins), which are transmembrane proteins forming complex membrane structures, called urothelial plaques. The urothelial plaques have been demonstrated to play a role in not only preventing the umbrella cells from dissociation of cell-cell conjunction, but also from bacterial adhesion, invasion, and dissemination [28]. The UPK family is formed of five proteins, uroplakin1a, 1b, 2, 3a, and 3b, which are assembled in the umbrella cells to build the urothelial plaques at the apical membrane. Our data presented here (Fig 3) suggest that four members of the protein family localize specifically to the umbrella cells, while one, uroplakin1b, is present in the whole urothelium.

### Proteins localized in intermediate/basal cells

**Fig 4:**
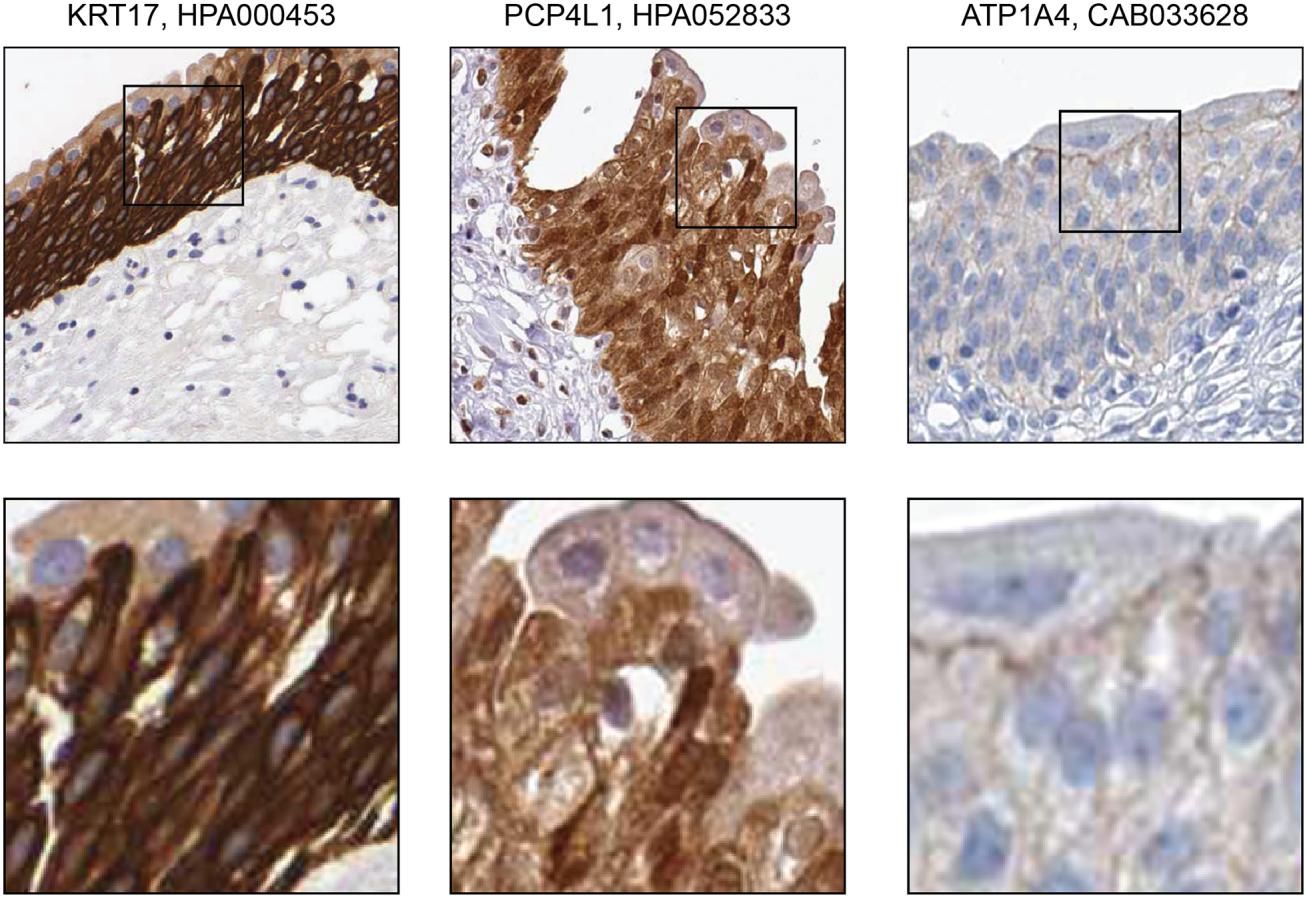
Immunohistochemistry-based protein profiling of elevated genes in urinary bladder that are expressed in intermediate/basal layer cells. Of the 90 elevated genes in urinary bladder, KRT17 (tissue enhanced gene), PCP4L1 (tissue enhanced gene) and ATP1A4 (tissue enhanced gene) are localize specificity in intermediate/basal layer cells in urothelium. All images are from the Human Protein Atlas. Titles on each images are gene and antibody names of concerned proteins.

The intermediate cells have a cuboidal to low columnar shape, oval nuclei with stippled chromatin, moderate amounts of cytoplasm and well-defined cell membranes. The basal layer of urothelium is made up of cuboidal cells, resting on basal lamina. The intermediate cells serve as superficial progenitors of the umbrella cells in adults, on the other hand, the basal cells are not the progenitors of intermediate or superficial cells in the developing urothelium and are likely to arise from a distinct progenitor population [29]. Out of the 90 genes with elevated expression in the urinary bladder, three were identified in the intermediate/basal cells (Fig 4). KRT17 has been described previously as expressed in urothelium and as activated through hypomethylation at the promotor in bladder cancer [30] and here we show a distinct localization to the intermediate/ basal cells. Similarly, PCP4L1 and ATP1A4, not previously described in the context of the urothelium, are both localized to the basal/intermediate cells. PCP4L1 has been described as a latent calmodulin inhibitor regulated by post-translational modification and/or co-factor interactions [31]. ATP1A4 has been described as expressed in male germ cells in testis, which agrees with our transcriptome data, and plays an important role in sperm motility [32].

### Analysis of proteins previously described as localized to the urothelium

**Fig 5:**
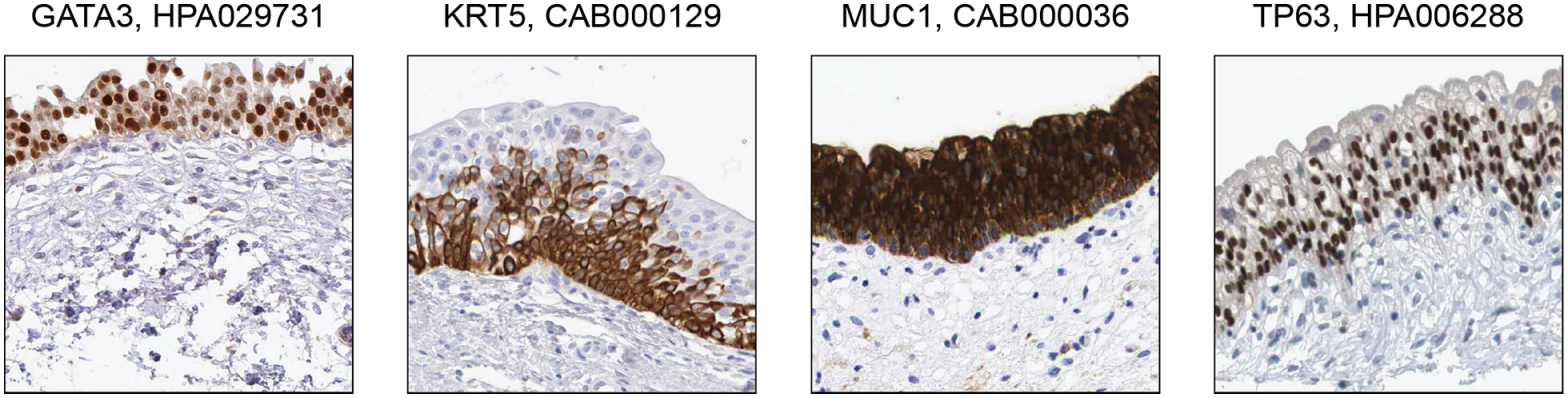
Examples of reverse analysis of proteins previously characterised as localized in urothelium. Four proteins selected based on literature (GATA3, KRT5, MUC1, TP63) are stained in urothelium and agreed with literature: GATA3 and MUC1 in urothelium and KRT5 and TP63 in intermediate/basal cells. All images are from the Human Protein Atlas and the title of each image show the respective gene and antibody names.

Interestingly, 61 of the 90 elevated genes have not been previously described in the context of urinary bladder and these are obvious targets for more in-depth studies. On the other hand, four proteins, KRT, TP63, GATA3 and MUC1, previously described as urothelium specific proteins [33] [34] [35], were not identified as elevated in the urinary bladder in our transcriptomics analysis. However, the immunohistochemistry analysis of these four proteins confirmed that these proteins are present in the urothelium (Fig 5) with KRT and TP63 located to the intermediate and basal cells of the urothelium, well in agreement with a previous study [33]. Expression of GATA3 and MUC1 was located to the entire urothelium, also in agreement with previous studies [34] [35]. Our data thus confirm their presence in urinary bladder, but our analysis suggests that there are other tissues with higher expression as compared to the expression in the urinary bladder. KRT5 show high expression in the esophagus and skin, while TP63, GATA3 and MUC1 show high expression in many other tissues, more specifically 8, 18 and 24 tissues, respectively. Thus, these proteins are here not defined as elevated in urinary bladder using our strict criteria for urinary bladder specificity.

## Discussion

Human gene expression profiling in tissues may provide useful information for understanding the physiology of the tissues and their diseases, as well as for discovery of novel biomarkers and tissue-specific drug targets. Here, we have used an integrated omics approach involving transcriptomics and antibody-based profiling to define the transcriptome and proteome of the urinary bladder. The analysis showed that expression of altogether 14,000 genes could be detected in urinary bladder and this is similar to the number of genes detected also in other tissues [7, 8]. Out of these, only 90 genes were found to have an elevated expression in the urinary bladder. Our RNA-seq analysis is based on urinary bladder tissue from only two individuals, but the high correlation in mRNA profiles between individuals in general [7] as well as between the two bladder samples (Fig 1) suggest low individual variation. However, for follow-up studies using the same approach to study the urinary bladder proteome in different pathological conditions, analysis of larger sample numbers is likely to be necessary to accommodate for known disease heterogeneity between individuals.

Furthermore, the spatial resolution was further investigated using immunohistochemistry to allow for localization of the corresponding proteins in the different subcompartments of the urinary bladder. 20 proteins were found to be localized to the entire urothelium, four to the umbrella cells and three to the intermediate/basal cells in the urothelium. Importantly, this analysis disclosed novel proteins not yet described in the context of the urinary bladder. Among them GREM1, BMP3, HOXA1 and SHH, which may play important roles in the urinary bladder development as suggested by previous reports showing these proteins’ involvement in differentiation [26].

Uroplakin 2 (UPK2) transcript was the most highly expressed in the urinary bladder and this protein was, as expected, localized to the umbrella cells [28]. Several other members of the uroplakin superfamily were also found to be elevated in the urinary bladder in the antibody-based profiling confirmed that also these proteins were localized to the umbrella cells. Interestingly, uroplakin 1B was present not only in the umbrella cells, but also in the entire urothelium. The difference in localization pattern of uroplakin 1B from other uroplakin proteins may suggest a unique function of uroplakin 1B as compared to the other uroplakin superfamily proteins.

In conclusion, our analysis identified genes of interest for the biology of the urinary bladder and provided lists of genes with enriched expression in different regions of the urinary bladder. This could prove to be a useful tool for both basic and clinical urinary bladder research.

## Supporting Information

S1 Table All urinary bladder elevated genes. (XLSX)

S2 Table The 23 group enriched genes. (XLSX)
